# Towards implementation of precision medicine biomarkers in early detection and prognostication of prostate cancer

**DOI:** 10.1007/s10555-026-10357-8

**Published:** 2026-07-23

**Authors:** Angela Yee, Jim Smith, Rajiv Kumar, Akash Sali, Euan J. Rodger, Aniruddha Chatterjee

**Affiliations:** 1 Department of Pathology and Molecular Medicine, Faculty of Medicine, University of Otago, Dunedin, New Zealand; 2 Department of General Surgery, Dunedin Hospital, Te Whatu Ora Health NZ - Southern, Dunedin, New Zealand; 3 Honorary Professor, SoHST, UPES University, Dehradun, India; 4 Department of Medicine, Faculty of Medicine, University of Otago, Dunedin, New Zealand

**Keywords:** Prostate cancer, Precision medicine, Genomic testing, Biomarkers, Liquid biopsy, Next-generation sequencing

## Abstract

Prostate cancer is the second-highest cause of cancer-related incidence and the fifth-highest cause of cancer mortality in males. Prostate cancer is a heterogeneous disease with a wide spectrum of clinical behaviour, ranging from indolent to highly aggressive. Molecular approaches, such as genomic testing, can augment existing clinical risk stratifications and tailor management to the individual. Genomic tests that sample biopsy or surgical tissue can provide a molecular risk assessment and identify actionable therapy targets. Liquid biopsy, while still emerging, may provide a non-invasive alternative to tissue tests and enable longitudinal monitoring of tumour status. We first discuss the molecular landscape of prostate cancer, before providing a detailed overview of the molecular approaches available for early detection and prognostication. Furthermore, methodological considerations and barriers towards clinical implementation for these tests are discussed, highlighting areas of future research.

## Introduction

Prostate cancer (PCa) is the second most diagnosed cancer and the fifth leading cause of cancer-related mortality in males worldwide [1]. The clinical trajectory of PCa is widely heterogeneous, which, with the limitations of modern diagnostic and prognostic approaches, creates substantial challenges for clinical management. A large proportion of PCa diagnoses represents the ‘overdiagnosis’ of indolent, low-risk disease, for which patients will never benefit from active treatment. Conversely, limitations in diagnostic accuracy still result in missed cases of ‘clinically significant’ PCa (csPCa), which represents PCa with a higher risk of subsequent morbidity or mortality. Defining PCa as truly clinically significant remains difficult, as evidenced by the continual evolution and variability of these definitions. Internationally accepted guidelines from the European Association of Urology (EAU) and the American Urological Association (AUA) define clinical risk groups for PCa based on data from a range of diagnostic investigations, including clinical tumour stage, serum prostate-specific antigen (PSA) values, histopathological variables, and radiological imaging. A large global disparity exists between PCa incidence and PCa-specific mortality, reflecting high rates of low-grade indolent PCa diagnoses, which are unlikely to benefit from radical curative treatment [2–5]. Regardless, the prognosis of PCa can be highly heterogeneous, and even non-fatal disease can result in significant morbidity. The cancer diagnosis itself can present a psychosocial burden for patients and an economic burden for global health systems. Curative treatment options for localised PCa also expose patients to procedure-associated adverse effects such as sexual dysfunction and urinary incontinence. Hence, the goal is to accurately distinguish between indolent PCa and aggressive csPCa, to guide curative therapy where appropriate and avoid needless overtreatment [6].

Modern PCa diagnosis follows a multi-faceted workflow, each component of which provides some level of prognostic information. The PSA test has long served as the standard screening test for PCa; however, it is limited by a lack of specificity, with the potential for overdetection of indolent PCa [7, 8]. Prostate magnetic resonance imaging (MRI) improves the selection of patients to undergo prostate biopsy and subsequently enables targeted sampling of suspicious lesions, increasing the detection of csPCa and reducing overdiagnosis. The Prostate Imaging-Reporting and Data System (PI-RADS) score standardises MRI interpretation and stratifies suspicious lesions on a 1–5 risk scale for the likelihood of csPCa [9]. Where there is suspicion of csPCa, a needle core prostate biopsy is performed for histopathological diagnosis. Historically, systematic prostate biopsy has been performed under ultrasound guidance, sampling uniformly through the prostate gland. Systematic biopsy can miss up to 37% of patients with csPCa; however, with the addition of pre-biopsy MRI-based stratification, that risk has been reduced to 28% [10]. Advances in biopsy technique, through a shift to targeted and peri-lesional biopsy, as well as increasing use of a transperineal approach, have further increased the accuracy of csPCa detection. Irrespectively, misprognostication can still occur if the biopsy sample is not representative of the tumour bulk [11]. Gleason score and International Society of Urological Pathology (ISUP) grade group, obtained through the histopathological verification of tissue, provide the most useful prognostic indicators for adverse PCa-associated outcomes, but cannot inform the full prognostic picture [11–15]. Hence, there is an ongoing push to develop novel risk classifiers that account for the inherent heterogeneity of PCa and have higher utility for predicting clinically meaningful endpoints.

**Fig. 1:**
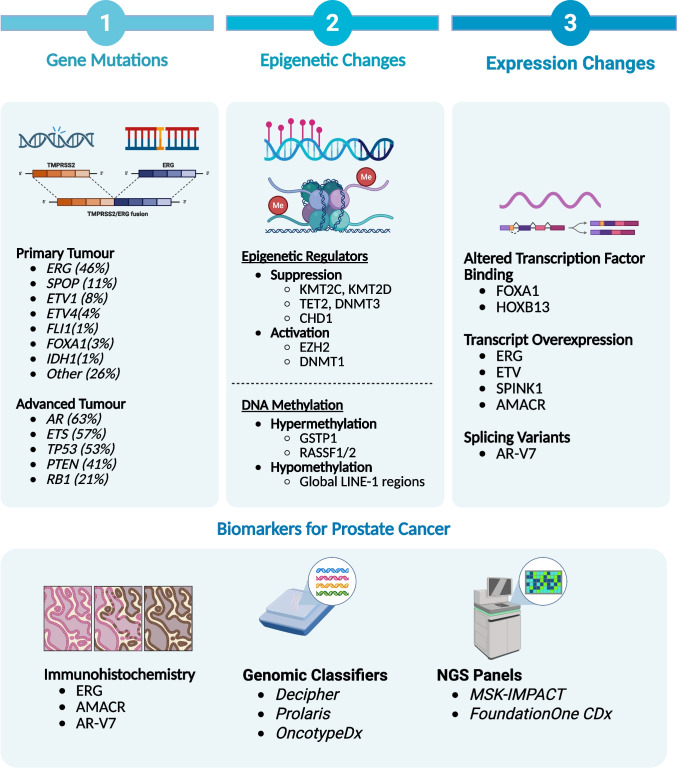
Genomic, epigenomic, and transcriptomic alterations in prostate cancer and currently available molecular biomarkers. Molecular subtypes can help to inform clinical subgroups which may require risk reclassification, experience earlier recurrence or metastasis, or benefit from targeted therapies

Molecular classification systems incorporating a range of omics data have been explored for this purpose over recent years. PCa is a complex disease characterised by a plethora of molecular alterations that drive tumour initiation, progression, and metastasis (Fig. 1) [16]. Precision oncology is a clinical approach that analyses the unique molecular features of a tumour to provide clinically relevant prognostic information and predict treatment response. The molecular signatures derived are cancer-specific and patient-specific, which facilitates informed clinical management, tailored to the individual patient. Genomic subclassifications have been popularised in different cancer types previously. In PCa, a number of putative molecular subtypes have been put forth; however, there is a lack of evidence to support their prognostic significance. Instead, tissue-based gene classifiers that prognosticate risk using gene expression signatures have amassed a growing body of evidence. Non-invasive ‘liquid biopsy’ sampling of molecular signatures in biofluids, such as serum, plasma, or urine, is a recent development in precision oncology and a rapidly progressing field. Liquid biopsy holds potential utility for non-invasive detection, real-time monitoring of tumour status, and tracking of molecular tumour progression events. This review will discuss the PCa molecular landscape, precision medicine approaches to detection and prognostication, and the challenges in implementing these approaches in both early and advanced PCa.

## The molecular landscape of prostate cancer

### Few mutations are conserved across primary prostate tumours

Compared to other cancers, patients with PCa share fewer genomic alterations [17]. The most highly conserved genetic aberration, found in 40–50% of PCa, is a gene fusion between the *TMPRSS2* and *ERG* genes, resulting in the overexpression of the ERG transcription factor [18, 19]. Alterations in *ERG* are frequently found in early PCa and lead to tumour development from a premalignant precursor lesion, prostatic intraepithelial neoplasia (PIN). Early ERG expression drives malignant transformation by rendering preneoplastic prostate cells susceptible to precipitating mutational events [20]. *TMPRSS2/ERG* is frequently associated with the deletion of the *PTEN* tumour suppressor gene and aberrant activity of the EZH2 histone methyltransferase, characterising a molecular subtype [21, 22]. This subtype can be characterised through the detection of *TMPRSS2/ERG* fusions with fluorescence *in situ* hybridisation (FISH) and genomic profiling or the detection of ERG overexpression with immunohistochemistry (IHC) and reverse transcriptase quantitative polymerase chain reaction (RT-qPCR). In the past, *TMPRSS2/ERG* has demonstrated considerable promise as a prognostic biomarker [23, 24], with potential utility in non-invasive urine sampling [25, 26]. However, recently, that has become controversial, owing to a failure to draw a link to clinical prognoses [27–30]. In their landmark study, *The Cancer Genome Atlas Network* clustered the genomic profiles of 333 primary PCa tumours, into seven genomic subtypes, characterised by genetic aberrations in *ERG* (46%), *ETV1* (8%), *ETV4* (4%), *FLI1* (1%), *SPOP* (11%), *FOXA1* (3%), *IDH1* (1%), and an unclustered 26% (Fig. 1) [16]. Under additional investigation, the tumour profile revealed a heterogeneous ‘long-tail’ of significantly mutated genes, with notable alterations in *TP53*, *KMT2C*, *KMT2D*, *AR*, *CDK12*, and *PTEN* [31]. Variation in the mutational landscape between cohorts of different ancestral lineages has also been captured by genome-wide studies. Asian cohorts report different sets of driver mutations from those in Western populations, including a 41% prevalence of *FOXA1* mutations and an 18% prevalence of *ZNF292* and *CHD1* deletions [32]. African-American patients harbour higher frequencies of *FOXA1*, *BRAF*, and *CHD1* mutations, as well as germline DNA damage repair (DDR) mutations, with increased involvement of the *MYC* oncogene pathway. Additionally, many mutations common in other cohorts (*ERG* fusion, *PTEN* loss) are less frequent in a cohort of patients with African ancestry [33–35]. Due to the heterogeneous genomic profiles of PCa, resulting from cross-cohort variation, it is challenging to identify a widely applicable clinical biomarker. Markers that do show promise, such as *TMPRSS2/ERG*, fail to translate due to a lack of prognostic significance. Research focus has, therefore, shifted towards the epigenomic and transcriptomic landscapes to identify markers that can explain shared clinical features.

### Conservation of epigenomic and transcriptomic features in primary prostate tumours

**Fig. 2:**
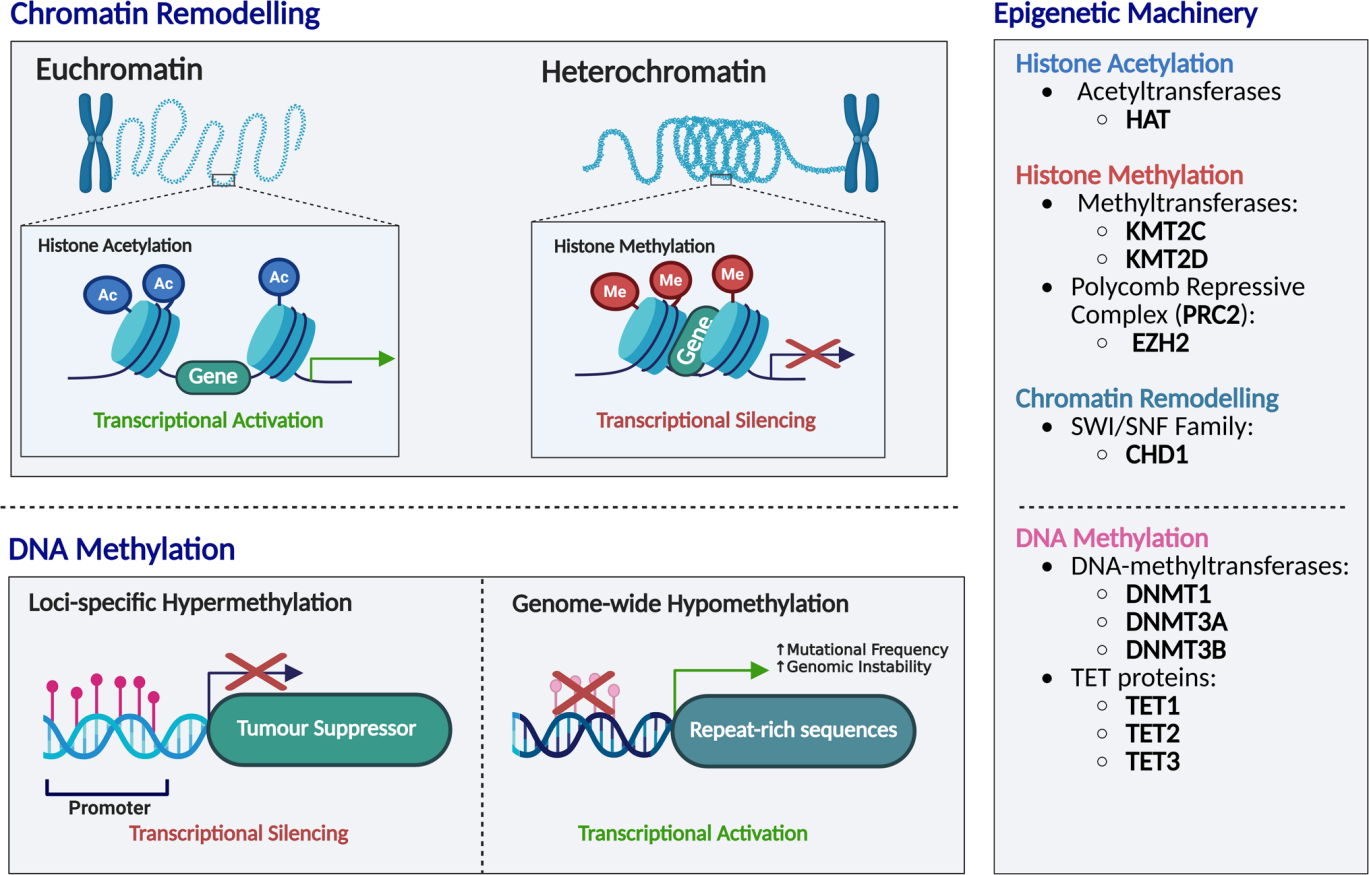
Epigenetic reprogramming in cancer: DNA methylation, chromatin remodelling, and transcriptional regulation. Histone acetyltransferases change the chemical structure of the chromatin from ‘open’ transcriptionally active euchromatin to ‘closed’ transcriptionally inactive heterochromatin, and histone methyltransferases perform the reverse action. Aberrant activity of these enzymes drives cancer-specific transcriptional programming. DNA methyltransferases add, while TET proteins remove methyl groups from DNA. Aberrant activity of these elements at promoters and other noncoding regions of the genome contributes to cancer-associated changes, such as decreased transcription of tumour suppressor genes and increased mutational frequency

Unlike mutations, epigenomic and transcriptomic changes are highly conserved across PCa, and the acquisition of these alterations contributes to tumour progression. Epigenetic mechanisms can modulate gene expression without causing changes to the DNA sequence, the most frequently studied of which is DNA methylation [36]. Mutations that affect epigenetic regulatory mechanisms are known to occur in approximately 20% of PCa, frequently affecting chromatin-remodelling enzymes (*EZH2*, *KMT2C*, and *KMT2D*), and DNA methyltransferases (*TET*, *DNMT1*, *DNMT3A*, and *DNMT3B*) [31, 37, 38]. In cancer cells, DNA methylation follows two general patterns: decreased methylation in repeat-rich regions of the genome, resulting in genomic instability—predisposing the cell to the accumulation of subsequent oncogenic mutations; and increased methylation in specific promoter regions of tumour suppressor genes leading to transcriptional silencing. Both mechanisms act in concert to promote uncontrolled tumour growth and progression (Fig. 2) [39]. Genome-wide methylation profiling in PCa has revealed aberrant methylation patterns, enriched within regions typically repressed by polycomb proteins, as well as regions associated with the androgen receptor (AR) gene interactions, and *MYC* oncogene binding [40]. Promoter hypermethylation of *GSTP1*, a member of the glutathione-S-transferase proteins involved in the DNA damage response, is the most commonly identified epigenetic alteration in PCa, and is associated with increased *DNMT1* activity [41]. *GSTP1* is silenced in PCa and in its precursor PIN, but not in the normal prostate or in benign prostatic hyperplasia (BPH) tissue [42, 43]. The presence in PIN suggests that its silencing is an early event in prostate tumourigenesis, and has led to investigations as a liquid biopsy marker for use in early diagnostic and prognostic contexts [44, 45]. The histone methyltransferase EZH2 is highly enriched in PCa—acting synergistically with DNA methylation to silence transcription, by recruiting DNA methyltransferases to specific gene promoters [22, 46, 47]. Dysregulation of chromosomal organisation has also been implicated in PCa progression. Chromatin remodelling enzymes dynamically regulate the chemical structure of the chromatin between ‘open’ transcriptionally active euchromatin and ‘closed’ transcriptionally inactive heterochromatin states (Fig. 2). A global reduction of transcriptionally repressive heterochromatin in PCa promotes genomic instability and is associated with poorer disease outcomes [48]. Altered chromatin states in late-replicating regions of the genome can increase the risk of chromosomal rearrangements, leading to gene fusions such as *TMPRSS2/ERG* [49]. Integrative epigenomic analyses have identified that the presence of AR and chromatin marks H3K27ac and H3K4me3 is associated with active transcription states in PCa [50]. CHD1, a tumour-suppressive chromatin remodeller, is lost in ~ 15% of primary PCa, resulting in redistribution of *AR*, followed by transcription and subsequent activation of pro-oncogenic pathways [51]. High-grade primary tumours exhibit features of epigenetic reprogramming, identified by altered chromatin organisation and increased levels of the transcription factors FOXA1 and HOXB13, which govern a common oncogenic transcriptional programme (Fig. 1)[52]. ERG overexpression can directly activate this transcriptional pathway by co-opting these two factors, leading to activation of the Notch signalling pathway [53, 54]. Distinct epigenomic and transcriptomic changes occur in prostate tumours that have progressed to advanced phenotypes, many of which are not found in primary PCa.

### Epigenomic and transcriptomic alterations in advanced prostate tumours

Advanced prostate tumours display molecular features that are distinct from localised primary tumours, driven by genomic instability and the progressive accumulation of oncogenic changes. Tumour growth in metastatic PCa is typically androgen-driven, thus initially termed metastatic hormone-sensitive PCa (mHSPC). These tumours tend to respond well to treatments which suppress androgen activity (androgen deprivation therapy; ADT). Over time, however, almost all ADT-treated tumours will eventually develop acquired resistance to androgen-suppressing agents and are then referred to as metastatic castration-resistant prostate cancer (mCRPC). Advanced PCa can develop through androgen receptor (AR)-dependent mechanisms where mutations or epigenetic changes drive aberrant AR signalling, as well as AR-independent mechanisms, such as the acquisition of aggressive molecular subtypes under therapy-driven selection pressures [55]. The genomic landscape of advanced PCa is enriched for alterations in the TP53 tumour suppressor gene and the AR gene, which suggests that 60% of advanced tumours may express a form of AR-dependency [37]. AR-dependent tumours can gradually become AR-independent through epigenetic and transcriptionally mediated mechanisms. For example, the loss of tumour suppressor genes TP53 and RB1 promotes a phenotypic shift from AR-dependent luminal epithelial cells to AR-independent basal-like cells [56]. Consistent with this epigenetically mediated phenotypic shift, AR-independent PCa activates a different transcriptional programme from AR-dependent PCa, notably exhibiting histone H3K4 methylation and increased FOXA1 transcription factor binding at the enhancer of *UBE2C*, a cell-cycle regulator and proto-oncogene [57].

Epigenetic changes, such as DNA methylation, often accompany transcription and phenotypic shifts [58]. In PCa, global DNA hypomethylation occurs later in the disease course than focal DNA hypermethylation. Specifically, mCRPC exhibits extensive global hypomethylation compared to primary PCa, particularly at *LINE-1* repeat elements [59, 60]. Approximately 22% of mCRPC tumours can be classified into a distinct epigenomic subtype, characterised by DNA hypermethylation-associated gene silencing in the *TET2*, *DNMT3B*, *IDH1*, and *BRAF* gene promoters [60]. Epigenetic elements underpin AR-independent mechanisms such as lineage plasticity [61] and transformation to the aggressive neuroendocrine prostate cancer (NEPC) subtype is a well-documented consequence. In NEPC and other aggressive subtypes, loss of TP53 and RB1 is accompanied by increased expression of the epigenetic reprogramming factors SOX2 and EZH2 [56, 62]. EZH2, in its phosphorylated form, interacts with the AR gene to alter chromatin states and reprogramme AR transcription, allowing tumour cells to enter a stem cell-like and neuronal cell-like ‘lineage-infidelity’ state [63]. Many of these molecular alterations will be able to inform the development of tumour-specific, treatment-specific, or metastasis-specific markers with translational potential.

## Utilities of precision medicine biomarkers in prostate cancer

### Early detection of clinically significant and high-grade disease

Biomarkers that can differentiate PCa states, especially ones that can be tested non-invasively, are highly valuable in the current landscape of PCa diagnosis (Table 1). Currently, serum PSA and prostate MRI are the two core tenets of the PCa early detection pathway before biopsy. However, the PSA test lacks specificity for PCa and is elevated in non-malignant prostate pathologies.

**Table 1:** Performance metrics for clinically relevant biomarkers. This table provides a broad overview of existing PCa biomarker tests. Many of these tests have different thresholds and have been modelled within different clinical contexts. AUC: Area under the hierarchical summary receiver operator characteristic curve

Biomarker-based test	Sample type	Description	Performance metrics	References
Utility: detection of early csPCa				
PSA	Serum	PSA	Sensitivity, 93%; specificity, 20%; AUC, 0.72	[7]
*Select-MDx*	Urine	2 gene panel: (DLX1, HOXC6)	Sensitivity, 91%; specificity, 36%; AUC, 0.86	[64]
*Exo-Dx*	Urine	2 gene exosome panel: (PCA3, TMPRSS2-ERG)	Sensitivity, 92%; specificity, 34%; AUC, 0.77	[65]
*MyProstateScore2*	Urine	18-gene panel: (TMPRSS2-ERG, SCHLAP1, OR51E2, APOC1, PCAT14, CAMKK2, PCA3, NKAIN1, B3GNT6, TFF3, SPON2, PCGEM1, TRGV9, TMSB15A, ERG, KLK4, HOXC6, KLK3)	Sensitivity, 94%; specificity, 54%; AUC, 0.81	[66]
Utility: prognostication of distant metastases in primary localised disease				
*Decipher*	Tissue	22 gene set (LASP1, IQGAP3, NFIB, S1PR4, THBS2, ANO7, PCDH7, MYBPC1, EPPK1, TSBP, PBX1, NUSAP1, ZWILCH, UBE2C, CAMK2N1, RABGAP1, PCAT-32, GLYATL1P4/PCAT 80, TNFRSF19)	AUC, 0.67–0.89*	[67, 68]
*Oncotype Dx*	Tissue	12 cancer-related gene set (BGN, COL1A1, SFRP4, FLNC, GSN, GSTM2, TPM2, AZGP1, FAM13C1, KLK2, SRD5A2, TPX2)	AUC, 0.65–0.824*	[68, 69]
*Prolaris*	Tissue	31 cell cycle–related gene set	AUC, 0.90*	[68, 70]

^*^AUC derived from a model combining clinical features and the genomic test

**Fig. 3:**
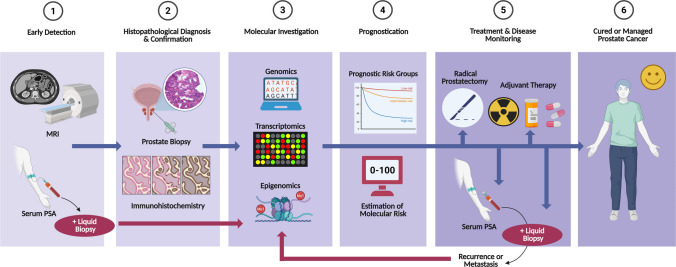
Opportunities for precision medicine in prostate cancer. A clinical workflow diagram of prostate cancer diagnosis, prognostication, and follow-up. Precision medicine biomarkers can be used in early detection, diagnostic confirmation, prognostication, and long-term disease monitoring

Liquid biopsy is a newly emerging field in PCa; it refers to the analysis of cell-free nucleic acids (cfDNA, cfRNA), circulating tumour cells (CTCs), or extracellular vesicles from biological fluids. In a healthy individual, cfDNA is regularly shed into biofluids as a result of cellular turnover. In a cancer setting, a fraction of these nucleic acids is shed from tumour cells, which are then referred to as circulating tumour DNA (ctDNA). A liquid biopsy approach enables longitudinal sampling of shed ctDNA to track molecular progression events, which is traditionally not feasible in the tissue medium due to limited sample availability and the invasive nature of repeated biopsy efforts. Early detection, disease monitoring, and assessment of therapeutic efficacy are all clinical niches that liquid biopsy markers could occupy (Fig. 3). Typically, advanced tumours shed more ctDNA, and specific ctDNA-detected alterations can be used to monitor advanced PCa [71–74]. All current commercially available ctDNA assays are NGS panels designed for advanced disease, as the amount of ctDNA shed in early disease is below the detection range for most of these assays. However, emerging studies highlight the promising potential for future applications in the early detection space, as it becomes more technologically feasible to reliably detect ctDNA in primary cancers with newer approaches [75, 76].

Urine and serum are particularly important biofluids for study in PCa. Urine markers have long been considered for PCa due to the anatomical course of the urethra, which passes through the prostate. A handful of well-characterised markers have been adapted for commercial usage: *PCA3* (*Progensa*), paired *DLX1* and *HOXC6* expression (*Select-MDx*), and paired exosomal *PCA3* and *TMPRSS2/ERG* expression (*Exosome Dx Prostate IntelliScore* (EPI)). Multigene panels, such as *MyProstateScore2* (MPS2), are one of the most recent developments. MPS2 is an 18-gene screening panel that assays for the presence of cfRNA transcripts in urine to inform a risk score assessing the likelihood of detecting high-grade PCa on biopsy [66]. These newer, multigene panels have begun to outperform previous commercialised tests [77]. Blood markers can be tested adjunctively in the serum alongside PSA in the screening and post-treatment settings. DNA methylation markers are promising liquid biopsy candidates due to their chemical stability, tissue specificity, and ability to capture information on both tissue of origin and tumour cell state, enabling sensitive cancer detection and disease monitoring [78]. Promisingly, cfDNA-based methylation markers have previously demonstrated an ability to discriminate between cancer and non-malignant status, as well as between localised and metastatic cancer, which may be useful in detecting patients with *de novo* metastatic disease [79, 80]. A promoter methylation blood panel testing for *GSTP1*, *RASSF1*, and *RASSF2* methylation has reported a higher specificity for PCa than PSA, justifying its potential value as an adjunct test [44]. Currently, there remains a lack of prospective randomised trials justifying the adoption of liquid biopsy markers into the clinic. Furthermore, standardised assays and guidelines for the use of these tests are not well established. Concentrations of ctDNA are typically low in biofluids, and insufficient input of cfDNA for typical polymerase chain reaction (PCR) or next-generation sequencing (NGS) assays can result in assay failure, accompanied by a nondiagnostic result.

### Prognostication in primary localised disease

High-risk molecular features, such as selected gene expression and DNA methylation signatures, have a long-held presence in the diagnostic biopsy setting. Immunohistochemistry is a cost-effective technique for assessing protein and gene expression, and it is accessible in most pathology laboratories. Alpha-methylacyl-CoA racemase (AMACR) is a reliable immunohistochemical marker that can differentiate between malignant and benign glands in prostate needle biopsy tissue [81]. Increased positivity for the AMACR and ETS-related gene (ERG) protein immunostains has been linked to higher grades of PCa, indicating potential prognostic significance [82]. DNA methylation has long been established for its ability to differentiate between tumour and non-malignant prostate tissue, especially in the case of glutathione S-transferase Pi 1 (GSTP1) gene promoter methylation [42, 43, 47, 83]. Further evidence suggests that methylation events can be used to distinguish between high and low-grade PCa [84]. The PCR-based ConfirmMDx commercial test assays for three high-risk promoter methylation markers in the GSTP1, Ras association domain family member 1 A (RASSF1A), and adenomatous polyposis coli (APC) genes, to guide re-sampling in patients where there is a suspicion of a non-representative sample from a high-risk tumour. However, it has very limited uptake as the evidence for this assay is not well established. In general, techniques to assay DNA methylation in tissue are underdeveloped in the clinical setting, with many laboratories being under-equipped to conduct those investigations. Thus, investigation of DNA methylation is restricted to the use of specialised commercial tissue panels, which are costly, limited in access, and most importantly, have limited prognostic potential. Immunohistochemical markers are more accessible and have a higher potential for rapid, global uptake; however, reporting guidelines require careful design so as not to contribute to the overdiagnosis of non-clinically significant disease. Gradual maturation in the design of prognostic biomarker studies has led to the creation of improved tissue biomarker panels (Table 1). In localised primary PCa, clinical recurrence (CR), distant metastasis (DM), and PCa-specific mortality (PCSM) are key prognostic outcomes that hold significant implications for clinical management. Currently, histopathological verification and grading of tumour tissue provide the most useful prognostic indicators, but it is not fully informative due to heterogeneity in clinical courses across the same grade of disease [11–13]. Localised, csPCa is treated first-line with either a radical prostatectomy (RP) or radiotherapy (RT). A selection of these patients will have their cancer recur and progress after initial radical treatment. Often, clinical recurrence is associated with a serum PSA rise above a determined threshold after treatment-associated disease remission, referred to as biochemical recurrence (BCR). Although BCR holds some associations with cancer outcomes, it is not always reflective of the true risk of DM or PCSM, and is considered an imperfect endpoint for prognostic model building [85]. Similarly, overall survival (OS), although clinically relevant, is often less useful in PCa, as many patients may be burdened by other comorbidities and die before PCa has progressed to a state able to cause mortality. It also fails to capture the potential morbidity associated with advanced PCa, even if not life-limiting [86]. Hence, there is an ongoing push to develop novel risk classifiers that account for the inherent heterogeneity of PCa and have a higher ability to predict clinically meaningful endpoints.

Prognostic models built with biomarkers, or genomic classifiers (GC), outperform traditional clinical-only classifier models [68, 87–90]. GCs test for the presence of a panel of markers in biopsy or RP tumour tissue samples to derive a risk score for adverse PCa outcomes. Observational studies based on commercial GCs have shown that testing is associated with a higher likelihood of conservative management, supporting the role for these tools in treatment de-escalation and prevention of overtreatment [91]. *Prolaris*, or the Cell Cycle Progression (CCP) Score, is a 31-gene expression panel designed to predict BCR and OM. The parent study retrospectively assessed a reported set of markers with reverse transcriptase quantitative polymerase chain reaction (RT-qPCR) on archival tissues [70]. Multigene panels, like *Prolaris*, improve the prediction of disease upstaging in patients with low-grade (ISUP 1) disease over singular biomarkers, like *TMPRSS2/ERG* [27]. The prostate *Oncotype Dx*, or the Genomic Prostate Score (GPS), is a 12-gene expression classifier designed to predict imaging-based clinical recurrence whose parent study determined a signature from public data before assessing it retrospectively with RT-qPCR in RP and matched biopsy tissue [69]. *Oncotype Dx* can reclassify 25% of patients to a different risk category, tending to counsel patients towards conservative management rather than active treatment in localised disease [92, 93]. *Decipher*, or the Genomic Classifier (GC) score, is a 22-gene expression classifier derived from RNA tissue microarray sequencing data, whose main strengths lie in its prospective study design [67]. Prognostic performances differ across the three GC models, but the quality of evidence for the *Decipher* model is superior to that of its counterparts [68, 94, 95]. There is poor inter-model correlation, highlighting the lack of gene set overlap and reproducibility due to variations in genomic methods and analysis pipelines [95]. *Decipher*’s evidence advantage may be explained by endpoint selection and a prospective study design. *Decipher* uses the model endpoint of progression to DM, which is more reflective of long-term outcomes compared to BCR, and uses prospectively generated gene expression data. This ensures that all potential confounders are controlled for by the same source that profiles, analyses, and designs the marker set. As tissue genomic testing necessitates a prior biopsy, researchers have begun to explore the possibility of non-invasive biofluid testing.

At present, the study of prognostic liquid biopsy biomarkers is preliminary at best. Previously, PCA3 has been investigated in the biofluids of patients treated with RP, but its prognostic ability is controversial, demonstrating poor accuracy for predicting adverse pathological features after radical prostatectomy in some studies [96, 97]. In a clinical trial of patients with undetectable PSA post-RP, increased numbers of circulating tumour cells (CTCs) were frequently detected. Their presence was associated with increased risk of BCR, suggesting that rising levels of CTCs precede the rise in PSA during prostate cancer recurrence [98]. Increased concentrations and a higher mutational burden detected on ctDNA are associated with early BCR, as suggested in other cancers [99, 100]. A few observational studies have indicated that this could also be the case in PCa [101, 102]. Recent trial evidence supplies a promising case for detectable ctDNA fraction as a marker. Although more data, especially on test parameters, is required before an attempt could be made at translation [71]. Many of these markers become increasingly relevant in an advanced disease context towards the evaluation and prediction of therapeutic response.

### Prognostication of early progression on androgen deprivation therapy

Molecular markers can be used as indicators of prospective therapeutic efficacy in PCa, particularly where it has recurred after first-line therapy (Fig. 3). Metastatic and recurrent PCa management often includes the use of long-term ADT administration. Subsequent progression of the tumour over time is common, which manifests as CRPC. *RB1* is a key mutational marker linked to earlier progression on ADT and poorer overall survival [103, 104]. The androgen receptor splice variant 7 (AR-V7) is reportedly associated with early BCR and early progression on ADT, and can be detectable in CTCs [29, 105–108]. There are also prognostic gene panels, such as PAM50. PAM50 is a 50-gene expression genomic classifier originally developed in breast cancer, which subtypes tumours into cell-type-based lineages. Prognostic correlations to the three PAM50 subtypes: basal, luminal A, and luminal B, have been identified in PCa. Luminal A tumours have the best overall prognosis, followed by luminal B, and basal-like tumours with the worst prognostic outlook. Basal-like tumours harbour higher numbers of *TP53* and *RB1* alterations, and are more likely to progress despite ADT and chemotherapy, with a larger proportion of patients in this subgroup experiencing BCR, DM, PCSM, and OM outcomes at 10 years [109–111]. Luminal B tumours are associated with increased *AR* expression and improved response to adjuvant ADT administered postoperatively [111, 112]. In blood, the ctDNA fraction and ctDNA-based somatic alterations can be tested for as prognostic factors. A high ctDNA fraction is linked to shorter time to ADT treatment failure [113]. *TP53*, *AR*, and *RB1* alterations in ctDNA predict poorer overall survival in mCRPC [113]. Management of advanced castration-resistant and metastatic disease is complex. As none of the aforementioned markers or assays has sufficient current evidence to demonstrate predictive value for guiding treatment selection, clinically their role remains prognostic.

### Targeted therapy selection in advanced disease

Disease-actionability markers can indicate the presence of molecular therapy targets and select patients for a tailored treatment regimen. The most clinically relevant disease actionability markers are mutations in DNA damage response (DDR) genes, which are responsible for various DNA repair pathways. These mutations have been characterised in approximately 19–20% of primary PCa, and this increases to 20–27% in mCRPC [16, 37, 114]. The homologous recombination repair (HRR) pathway is responsible for repair of double-stranded DNA breaks. Mutations in HRR, such as *BRCA1/2*, *CHEK2*, and *ATM*, are predictors of response to poly ADP-ribose polymerase (PARP) inhibitors [115]. The mismatch repair (MMR) system is responsible for correcting mutations in repeat DNA sequence tracts. Mutations in *MSH2*, *MSH6*, *MLH1*, and *PMS2* can inactivate the MMR system and cause microsatellite instability (MSI). MSI is a biomarker that can be used to guide immunotherapy selection, and has been reported in ~ 2 to 12% of mCRPC [37, 116]. NGS assays can identify hundreds of mutations with existing or potential prognostic and therapeutic utility. Mutations which are known to be clinically actionable can determine patient eligibility for various targeted therapies. As examples, the Memorial Sloan Kettering Cancer Centre (*MSK-IMPACT*) and *FoundationOne CDx* commercial NGS panels both assess more than 300 genes of known relevance in metastatic cancer to identify patients with susceptibility to PARP or immune checkpoint inhibitors [17, 117]. Liquid biopsy ctDNA NGS mutational panels are an innovation with promising potential applicability in advanced PCa. Recent preliminary studies of the *FoundationOne Liquid CDx* panel have reported an increased overall survival benefit after receiving therapy based on NGS panel counselling [118]. However, careful cost–benefit analyses should be undertaken before adoption of clinical NGS methods. The degree of targeted therapeutic actionability is dependent on cancer type. Currently, the range of available targeted therapies is smaller for PCa than for other cancers, and that is further constrained by regional access limitations. Despite being resource-intensive, genomic testing is invaluable for patients who would be potentially eligible for targeted therapies. Further emphasising their clinical value, the 2026 European Society for Medical Oncology (ESMO) guidelines now recommend routine genomic biomarker testing (*BRCA1*, *BRCA2*, *CDK12*, *PALB2*) for all patients diagnosed with CRPC, as well as situation-dependent germline and MMR deficiency testing [119].

## Barriers to clinical biomarker translation

### Molecular heterogeneity

Intratumoural heterogeneity stands as a major barrier in the discovery and interpretation of molecular markers [120]. PCa is characteristically multifocal and multiclonal, manifesting as multiple, separated focal areas of tumour composed of different clonal cell populations [121]. Extensive inter- and intra-individual genomic heterogeneity has been identified from the profiling of multifocal primary PCa, as 76% of patients do not have any common point mutations across their paired tumour foci [122]. Heterogeneity in DNA methylation and copy number alterations (CNAs) also suggests divergent evolution across clonal subpopulations of tumour cells [123]. In biomarker studies, this introduces the possibility of biased sampling and capture of tumour foci that are not true molecular representatives of the tumour bulk. Detection of tissue biomarkers is dependent on the tumour focus sampled; for example, multifocal ERG expression is present in only 16% of patients, where in most instances the transcript is expressed inconsistently in some tumour foci and not others [124]. Furthermore, a non-representative sample can result in a falsely negative test, leading to a missed diagnosis of csPCa, which can occur in up to one third of negative biopsies [10, 11]. More specifically, if the overall tumour is clinically significant (ISUP ≥ 2) while the biopsy sample only captures regions of low-grade (ISUP 1) disease, it could result in delays to curative treatment, or even missing an opportunity for cure entirely. Genomic tests on non-representative samples will exacerbate the Type II error, as the test may under-detect higher-risk molecular changes in adjacent cancer tissue, or miss these entirely [125]. Sampling dependency directly affects the accuracy of tissue GCs, increasing variability in assay results and reducing prognostic accuracy [126].

Solid PCa tumours are frequently mixed with non-malignant epithelial cells, as well as immune and stromal cells and fibroblasts from the tumour microenvironment. The molecular profiles of bulk tissue-based investigation will always contain a component of non-malignant cell signals, which is a potential confounder in molecular biomarker analysis. These molecular profiles can be resolved with single-cell sequencing technologies, but they are costly and resource-intensive, and thus would be difficult to implement in translational laboratories [127]. A similar caveat exists in the liquid medium, where cfDNA is composed of a mixture of ctDNA and background cfDNA, mainly from immune cells. Before translation, optimised methodologies to parse tumour signals from background non-malignant signals need to be developed to permit accurate and clinically useful biomarker determination.

The development of non-representative disease markers, driven by the historical underrepresentation of ethnic minorities within clinical trials and public genomic databases, can contribute to health outcome disparities when translated into the clinic [128, 129]. Known genomic variation exists across different ancestral groups [32–35]. As evidenced in a real-world application, the *Decipher* panel, built on a non-representative cohort of mainly European Americans, has displayed decreased prognostic performance when applied to African-American patients [130]. This could be further complicated by non-equitable access to healthcare resources. Molecular testing is inaccessible in many parts of the world, which results in a lack of molecular representation from health centres in countries with a low human development index (HDI) within public databases. Access to molecular testing is still limited to a wealthy few in affluent countries. Efforts still need to be made to bridge the gap in access and create equitable biomarkers that are reflective of the testing population.

### Assay standardisation

**Fig. 4:**
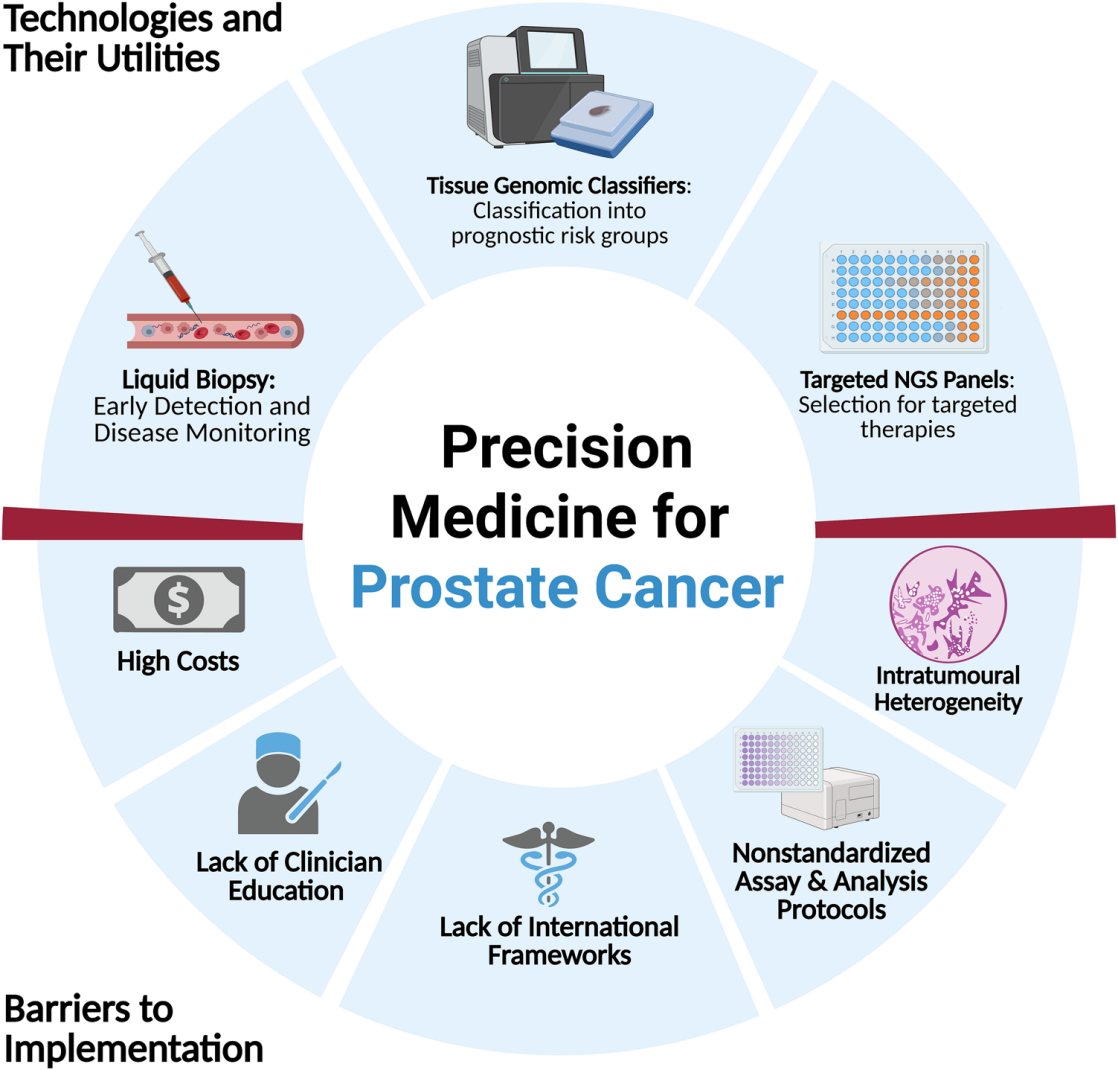
A schema of precision medicine biomarker utility and existing barriers to their clinical implementation

Standardised assays for genomic analyses have not been established in many clinical laboratories. Performing genomic analyses is time-, resource-, and personnel-intensive (Fig. 4). Testing may not always guarantee results, and assay failure rates range from 18 to 36% [131, 132]. Nucleic acid extraction failure is a typical issue, especially due to limited sample availability when testing biopsy tissues and biofluids. The sample preservation method can influence assay performance. Formalin-fixed paraffin-embedded tissue blocks are standard substrates for genomic analysis, but DNA degradation can occur during long-term storage, leading to test failure [133]. In one study assessing NGS-based mutational panel performance across sample types in PCa, they demonstrated that tissue samples performed the best, where all samples (100%) were able to yield mutational information. Comparatively, plasma and urine samples had moderate detection rates at 67.6% and 65.6%, respectively, while semen samples showed a lower detection rate of 33.3% [134]. This implies assay failure may occur in ~ 30% of plasma and urine samples, suggesting that NGS panels would be more accurate if tissue were tested alongside urine and plasma.

Clinicians have reported low confidence in the interpretation of test results, particularly from ctDNA, and agree that there is a lack of formal health professional training on the use of genomic testing [135]. However, there is currently no standardised reporting system for genomic analyses in both tissue and biofluids, which results in variation in regional practices. NGS panels are high-cost investigations, but clinically, their degree of benefit remains unknown due to variation in use in different countries, regions, and populations [136–138]. Many genomic assays have long testing turnaround times, which can delay diagnosis and treatment, waiting on information that may or may not be clinically relevant. Furthermore, even if the result is clinically relevant, oftentimes access to the relevant targeted treatment or an appropriate clinical trial may not be available [135]. These features must all be considered in the design of standard laboratory-to-clinical reporting guidelines.

### Development of implementation pathways

Many precision medicine biomarkers have an uncertain evidence base, resulting in only a mere handful of biomarker panels being integrated into national frameworks and disease guidelines (Fig. 4). The general finding has been that tissue GCs increase the likelihood of diagnosis and reclassification to low-grade disease in a post-biopsy setting, which implies a shift towards conservative management [92, 139]. However, these findings have been accompanied by a lack of certainty, as retrospective validation studies using archival tissues still outnumber prospective studies and randomised controlled trials in the overall evidence base [68, 91, 140]. Retrospective studies would inadequately reflect the modern performance of these tools, due to major changes to risk-stratification practices, histopathological grading, and biopsy technique in recent years. Furthermore, the context for their usage is frequently unclear, and only a few resources provide a clear decision-making pathway from biomarker testing to test interpretation and clinical management implications. Due to their lack of governing bodies and clinical infrastructure, there is also a lack of health education surrounding genomic tests. For clinicians, these tests are numerous and difficult to interpret, and without specialist and guideline-based assistance, they do not directly lead to a clinical decision. Inadequate education of healthcare providers can trickle down to the patient level, as many patients have reported misunderstandings and miscommunications on the role of these tests. Additionally, certain patients report being unaware that testing had occurred in the first place, highlighting a potential ethical issue surrounding informed consent [141]. Thus, the current consensus is that these biomarkers are unable to independently guide treatment decisions in PCa and must be used selectively with consideration of other clinical parameters [142, 143]. Conducting tests that would be unlikely to affect the treatment course will only result in increased economic burden on the healthcare system and potentially on patients. Without adequate structured guidelines or education, it is reasonable for many clinicians to remain cautious towards trialling these new tests.

Currently, genomic tests do not have an international set of standards they are held against, which are endorsed by an international health organisation. In some countries, national health organisations have begun to adopt frameworks which rank and define the clinical utilities of some of these tests. The creation of these frameworks would permit the establishment of clinical guidelines and support patient and clinician education on their usage. An existing framework that has been adapted for this purpose is the Simon hierarchy of evidence, which oversees tissue-based GC panels only. Simon et al. created a hierarchy of evidence to assess the quality of the evidence for archival tissue biomarker studies [144]. They suggest that clinical utility can be established with evidence from prospective studies that have been specifically designed and powered to address the tumour marker question, where the ideal samples would be sourced from randomised trials of cancer therapeutics or high-quality observational studies [144, 145]. The Simon hierarchy has been recently endorsed by the American National Cancer Centre Network (NCCN) [142]. In their 2024 guidelines, they reviewed the evidence base and assigned *Prolaris*, *Oncotype Dx*, and *Decipher* the Simon levels of evidence IIIC, IIIC, and IB, respectively, with *Decipher* ranking the highest. They also designed a set of decision-impact guidelines for *Decipher* score utility in second-line salvage therapy decision-making, with test results providing a direct laboratory-to-clinical connection. As evidenced in this example, an endorsed framework that oversees all NGS panels can create guidelines that assist with their interpretation, thus facilitating clinical translation. Further down the line, the creation of structured training and accreditation processes could promote clinician-side education and improve confidence in counselling patients for genomic testing.

## Conclusion and perspectives

Precision medicine biomarkers have strong potential to guide early detection, prognostication, and the selection of targeted therapies in PCa. Currently, they have experienced limited clinical translation through the dissemination of commercial panels, which are now supported by a reasonable body of clinical trial evidence. Liquid biopsy tests are emerging with promise to non-invasively interrogate tumour status, yet are lacking in clinical trial evidence and affected by persistent methodological limitations. Genomic testing and targeted NGS-based panels are recommended in mCRPC to assess therapeutic actionability, but are resource-intensive.

All precision oncology biomarkers face the challenges of cost, sampling variability, and unclear test interpretation (Fig. 4). The landscape is shifting towards a multiple-omic, or ‘multi-omic’ approach, as newer discovery studies look at data from multiple molecular modalities to create more complex biomarker panels that improve accuracy. This also heralded the curation of large comprehensive ‘omics’ databases containing genetic, transcriptomic, and epigenomic levels of data, which once analysed may yield signatures more robust than existing examples. Already, studies have suggested that singular biomarkers sourced from one modality are insufficiently informative of clinical prognosis [27, 30, 103]. Single or multi-omics tests that assay tens to hundreds of markers promise to improve marker detectability and prognostic performance [146–148]. Further study in prospective and randomised controlled trials can help form an increasingly solid evidence base for clinical translational efforts.

## Data Availability

No datasets were generated or analysed during the current study.

## Abbreviations

ADT: Androgen deprivation therapy
AR: Androgen receptor
AUA: American Urological Association
BCR: Biochemical recurrence
CfDNA: Cell-free DNA
CfRNA: Cell-free RNA
CR: Clinical recurrence
CRPC: Castration-resistant prostate cancer
csPCa: Clinically significant prostate cancer
CTC: Circulating tumour cell
CtDNA: Circulating tumour DNA
DM: Distant metastasis
EAU: European Association of Urology
FISH: Fluorescence *in situ* hybridisation
IHC: Immunohistochemistry
ISUP: International Society of Urological Pathology
mHSPC: Metastatic hormone-sensitive prostate cancer
MRI: Magnetic resonance imaging
NCCN: National Cancer Centre Network
NGS: Next-generation sequencing
OS: Overall survival
PCa: Prostate cancer
PCSM: Prostate cancer-specific mortality
PIN: Prostatic intraepithelial neoplasia
PI-RADS: Prostate imaging-reporting and data system
PSA: Prostate-specific antigen
RP: Radical prostatectomy
RT: Radiotherapy
RT-qPCR: Reverse transcriptase quantitative polymerase chain reaction
